# Attitudes of Students of Social Sciences and Humanities towards People with Physical Disabilities (MAS-PL)

**DOI:** 10.3390/ijerph19031544

**Published:** 2022-01-29

**Authors:** Dorota Tomczyszyn, Anna Pańczuk, Adam Szepeluk

**Affiliations:** 1 Department of Sociology, Faculty of Social Sciences and Humanities, Pope John Paul II State School of Higher Education in Biała Podlaska, 21-500 Biała Podlaska, Poland; 2 Department of Physiotherapy, Faculty of Health Sciences, Pope John Paul II State School of Higher Education in Biała Podlaska, 21-500 Biała Podlaska, Poland; a.panczuk@dydaktyka.pswbp.pl; 3 Department of Computer Science, Faculty of Technical Sciences, Pope John Paul II State School of Higher Education in Biała Podlaska, 21-500 Biała Podlaska, Poland; a.szepeluk@dydaktyka.pswbp.pl

**Keywords:** disability, attitudes, MAS-PL, students, Poland

## Abstract

Negative social attitudes towards people with disabilities are a serious barrier to their social, professional, and cultural functioning. Due to negative perception, disabled individuals are often homebound. The present study was an attempt to compare declared attitudes with the results of other studies using the Polish version of an international assessment tool. The aim was to verify the MAS-PL tool and to analyse the attitudes of students of social sciences and humanities towards people with physical disabilities. An additional aim was to compare declared attitudes with the results of other studies using the Polish version of an international assessment tool for students of medicine and health sciences. In total, 540 students were surveyed using the Polish adaptation of the Multidimensional Attitudes Scale towards Persons with Disabilities (MAS-PL). The study confirmed the high reliability of the MAS-PL Scale. The mean global score of the scale was 82.79. There were no significant differences in the global MAS-PL score depending on respondents’ sex; however, an effect of this variable on the subscales was found. The surveyed females exhibited more positive attitudes in terms of the cognitive and behavioural components, whereas the males showed more positive attitudes in the affective subscale. There was no significant impact of the place of residence, age, and majors. Along with the year of studies, the intensity of the global and affective scale slightly increased. The surveyed students of social sciences and humanities exhibited similar attitudes towards people with physical disabilities to those declared by Polish students of medical and health sciences surveyed by Radlińska et al. The authors recommend implementation of didactic classes, projects, and activities at the stage of planning and creation of student education programs to promote tolerance towards disabled people.

## 1. Introduction

There are over one billion people with disabilities in the world (approx. 15% of the world’s population), and this number is growing [1]. In Poland, people with disabilities accounted for 12.2% of the country’s population (almost 4.7 million), according to the last National Census of 2011 [2].

Due to the wide conceptual scope of the disability term, one universal definition of disability in the literature and in legal acts has never been developed. This concept cannot be clearly and easily defined but has undergone a noticeable evolution over the years. In European Union countries, several different terms are often used to define ‘disability,’ even in one country, and each country has its own disability assessment system [3].

In the 21st century, the approach to the disabled has changed radically, as evidenced by the documents of the WHO and the UN Convention on the Rights of Persons with Disabilities. Currently, the emphasis is placed on the potential and opportunities for the disabled to participate in social life rather than on their limitations exclusively. The social importance and the context of disability issues were presented in the International Classification of Functioning, Disability, and Health ICF, which was formulated in 2001 at the World Health Assembly. The presented social model of disability “sees the issue mainly as a socially created problem, and basically as a matter of the full integration of individuals into society. Disability is not an attribute of an individual, but rather a complex collection of conditions, many of which are created by the social environment. Hence, the management of the problem requires social action, and it is the collective responsibility of society at large to make the environmental modifications necessary for the full participation of people with disabilities in all areas of social life. The issue is therefore an attitudinal or ideological one requiring social change, which at the political level becomes a question of human rights” [4] (p. 20). As declared by the preamble of the Convention on the Rights of Persons with Disabilities, “recognising that disability is an evolving concept and that disability results from the interaction between persons with impairments and attitudinal and environmental barriers that hinders their full and effective participation in society on an equal basis with others” [5] (p. 2). The document is binding for establishment of the laws of the Member States. It emphasises that everyone, including people with disabilities, is equal before the law and should be included in the mainstream of social life (inclusion). It also highlights the consequences of social and environmental barriers to the everyday life of people with disabilities [5]. The Polish Parliament unanimously adopted the Convention on 15 June 2012 [6], the President of the Republic of Poland signed the document on 26 July 2012, and the Convention was finally ratified on 6 September 2012 [7].

Many obstacles faced by the disabled are generated by inappropriate social attitudes [8,9,10,11,12]. Severe architectural, economic, and educational barriers hinder the functioning of a disabled person, whereas social barriers resulting from indifferent or negative attitudes play a key role in development of the individual’s self-awareness and motivational sphere in the professional, social, and cultural space. Social perception of people with disabilities influences their personal choices, decisions, mindset, awareness, and separateness of ‘us’ and ‘them’. Negative social attitudes create prejudices, fears, and negative patterns of behaviour.

The attitude determines the permanent positive or negative assessment of people, objects, and concepts [13]. There are three components of social attitude: cognitive, affective-evaluative, and behavioural (functional). They are all interrelated and conditioned. The cognitive component is a range of ideas, i.e., knowledge with a varying degree of certainty, on the object of the attitude. The affective-evaluative component contains positive or negative feelings, which are the basis for the assessment of the object of the attitude. The behavioural component determines the predisposition to react (action or avoidance) in terms of updating or referring to a specific object of the attitude [14,15,16]. The most common attitudes towards people with disabilities are positive (acceptance) and negative (rejection or avoidance). They are influenced by a set of external and internal factors. External factors are the socio-cultural conditions, while internal factors result from personal experiences of a disabled person [13,14]. The following external and internal factors can be distinguished:

- factors related to the civilization development promoting the concept of recognition of the dignity of every human being;
- culturally conditioned factors, i.e., a system of norms, values, religion, traditions, customs, and legal norms acquired in the process of socialization of the individual;
- factors related to the broad-sense socio-economic system. The sense of security of one’s existence has a positive effect on attitudes towards people in need. Opportunities and changes in the economic situation may cause attitudes of avoidance or acceptance towards people with disabilities;
- factors related to the system of human values, e.g., attitudes of tolerance, acceptance of people with disabilities. The system of norms and patterns of behaviour, the hierarchy of values, and the possibility of manifesting one’s choices and feelings determine the personality of a person and his/her assessment of people and events.

In Poland, attitudes towards people with disabilities have been investigated. However, they were based mainly on tools used only in the country, and there are no analyses facilitating comparison of the results with international studies. Such possibilities are provided by the multidimensional tool (MAS) developed by Findler et al. for assessment of attitudes towards people with disabilities with the Polish adaptation proposed by Radlińska et al. [17,18]. This tool facilitates the comparison of results of studies carried out using the original English-language MAS scale and its other non-English adaptations. An additional advantage of this tool is the fact that it takes into account all three dimensions (components) of attitude: cognitive, affective, and behavioural. Furthermore, this tool is based on projection techniques. This solution reduces the risk of reporting false but expected and socially acceptable responses by the respondents [17].

The adaptation of the MAS scale to the Polish conditions and the first studies with the use of the tool in Poland were carried out in a very specific group of students of medical and health sciences [18,19]. As indicated by a literature review, medical students and health care professionals exhibit more positive attitudes towards people with physical disabilities than persons unrelated to the health care sector [20]. The authors of the present study decided to check the reliability of the MAS-PL scale in a group of students of social sciences and humanities and to analyse their attitudes towards people with physical disabilities. An additional aim was to compare declared attitudes with the results of other studies using the Polish version of an international assessment tool for students of medicine and health sciences.

## 2. Materials and Methods

The study, conducted in February and March 2021, involved 540 full-time students of social sciences and humanities at the Pope John Paul II State School of Higher Vocational Education in Biała Podlaska (eastern Poland). The online survey was anonymous and voluntary. The respondents did not receive any remuneration for participating in the study.

### 2.1. Questionnaire

The study was based on the use of the Polish adaptation of the questionnaire for assessment of attitudes towards the disabled: The Multidimensional Attitudes Scale towards Persons with Disabilities (MAS-PL). The Polish MAS scale adaptation was developed and published by Radlińska et al. [18]. Its structure corresponds to the original MAS scale developed by Findler, Vilchynski, and Werner [17].

As with the original MAS scale, the MAS-PL survey consists of three subscales corresponding to three attitude components: affective, cognitive, and behavioural. The scale comprises emotions (16 items), beliefs (10 items), and declared behaviours (8 items). Similar to the original MAS scale, the MAS-PL questionnaire begins with a vignette and a description of a scenario of a random and circumstances-forced encounter of a person in a wheelchair by a non-disabled person in a cafe. The respondent is expected to imagine this situation and indicate emotions, thoughts, and potential behaviours that may be elicited in the non-disabled person. Therefore, the respondent has the role of a narrator and is asked to deduce the attitude of the “main character”. The questions are not addressed directly to the respondent; instead, they are based on a projection mechanism, which ensures a higher degree of honesty in the answers. The use of the social scenario vignette helps the respondents to project their own emotions, thoughts, and behaviour onto the given situation. The five-point Likert scale of answers is used, where 1 means “not at all” and 5 means “very much” (reverse scoring is used for positive attitudes). A higher score means a more negative attitude towards people with disabilities [18].

In the survey, the respondents also provided information about the following socio-demographic data: sex, age, place of residence, study majors, and year of study. Due to the homogeneity of the group of students in terms of their education level, this socio-demographic variable was deliberately omitted in the analyses.

### 2.2. Statistical Analysis

The statistical analysis was performed with the use of STATISTICA v. 13.0 PL and Microsoft Office 2010. The MAS-PL questionnaire was verified with a factor analysis supplemented with Kaiser-normalised Varimax rotation for uncorrelated factors. Cronbach’s alpha coefficient was calculated to assess reliability. Descriptive statistics (arithmetic means, standard deviations, minimum, maximum, skewness, and kurtosis) were presented for each questionnaire subscale, and the comparison with regard to the selected socio-demographic variables was carried out using the non-parametric Mann-Whitney test and the Kruskal-Wallis U test. The normality of the distribution was checked with the Shapiro-Wilk test. Spearman’s rank-order correlation was used to analyse the relationships between ordinal and quantitative variables. In all analysed cases, the significance level of *p* < 0.05 was assumed.

## 3. Results

### 3.1. Socio-Demographic Characteristics

In total, 540 students of social sciences and humanities were surveyed. The largest groups were represented by females (67.8%) and residents of rural areas (51.3%). The mean age of the respondents was 21.6 ± 4.3 years; the youngest student was 18 and the oldest respondent was 49 years old (Table 1).

### 3.2. Verification of the Reliability of the MAS-PL Scale

**Figure 1:**
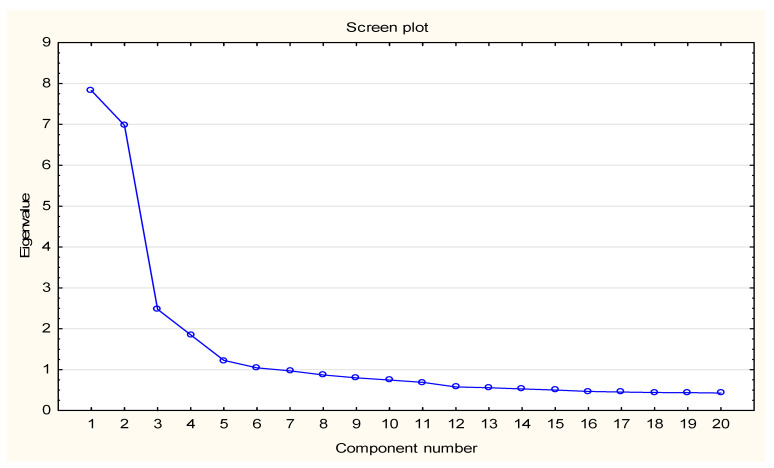
Scree plot of the three-factor MAS-PL structure.

The work on the questionnaire began with the determination of the number of individual subscales. To this end, factor analysis supplemented with Kaiser-normalised Varimax rotation for uncorrelated factors was used. In the study, six and three factors were assigned the total loading above 1 and above 2, respectively. The result of the analysis was a three-factor structure explaining in total over 50% of the observed variance, as confirmed by the screen plot (Figure 1). The structure is consistent with the original structure of the questionnaire, and the internal structures of all three subscales are similar to the original version. Only some items belonging to the emotional domain contained more than one factor, while the other items had from strong (>0.8) to weak (<0.3) factor loadings.

The analysis of the reliability of the individual subscales of the questionnaire revealed that each of the tested subscales had a reliability coefficient greater than α = 0.8, and the entire tool had a level of α = 0.881 (Table 2). These results prove the high reliability of the MAS-PL questionnaire.

### 3.3. Attitudes of the Surveyed Students towards Disabled People

The average number of points scored by the surveyed students in the global score of the MAS-PL scale was 82.79. The smallest global score was 39, while the largest value was 136. The distribution of the global score was clearly similar to the normal distribution typical for the general population, as evidenced by the non-significant result of the Shapiro-Wilk test (*p* = 0.375). The distributions of all three subscales differed significantly from the Gaussian distribution (*p* < 0.001). The kurtosis suggests the weakest concentration of scores around the means and the greatest differentiation of the responses in the affective subscale as well as the strongest concentration of scores around the means and the lowest differentiation in the behavioural subscale responses (Table 3). 

The statistical analysis revealed no significant correlations between the affective and cognitive subscales (*p* = 0.9620). In all other cases, there were statistically significant positive correlations between the individual subscales and between the subscales and the global MAS-PL score (Table 4).

Table 5 shows the MAS-PL results for the respondents representing different majors. The analysis did not reveal any statistically significant differences between the respondents representing the different majors and the global MAS-PL score (H = 7.46; *p* = 0.2804), the affective subscale (H = 10.11; *p* = 0.1202), the cognitive subscale (H = 10.74; *p* = 0.0969), and the behavioural subscale (H = 5.58; *p* = 0.4717) (Table 5).

### 3.4. Attitudes of the Respondents towards Disabled People versus Socio-Demographic Variables

The analysis of the effect of the socio-demographic variables was focused on the sex, place of residence, age, and year of study. No significant differences were found in the global score in the analysis of the effect of sex (Z = 0.85; *p* = 0.3948). In turn, there was a significant effect of sex on the three subscales of attitudes towards disabled people. In the case of the cognitive (Z = 3.64; *p* = 0.0003) and behavioural (Z = 3.09; *p* = 0.0020) subscales, the males scored significantly higher numbers of points (on average by 2.61 and 1.32, respectively), whereas the females scored higher values in the affective subscale (on average by 2.69; Z = −2.77; *p* = 0.0056) (Table 6).

Statistical analysis did not show any significant effect of the age variable on the levels of the global scale and individual subscales in MAS-PL. The intensity of the global and affective scales slightly increased with the year of study (r = 0.12; *p* = 0.0061, r = 0.19; *p* < 0.0001, respectively) (Table 7).

The analysis of the effect of the place of residence showed no significant impact of this variable on the results in the global scale and the subscales (Table 8).

## 4. Discussion

In Polish studies on attitudes towards people with disabilities, diagnostic survey methods were most often employed with questionnaires used only in the country. Therefore, it is difficult to compare their results. The research indicates that, at least at the declarative level, the respondents support integration attitudes [21,22,23,24,25,26].

The Polish adaptation of the Multidimensional Attitudes Scale towards Persons with Disabilities (MAS-PL) facilitates comparison of the results with international surveys. The analysis of the reliability of this tool carried out by Radlińska et al. indicated highly satisfactory results (α = 0.875). Similarly, the factors of the individual components of attitudes towards disabled people exhibited high measurement accuracy—each subscale achieved a reliability coefficient exceeding α = 0.8. This analysis was carried out on a group consisting mainly of students of medical and health sciences (the most numerous group comprised physiotherapy students) [18]. The present study assessed the reliability of the MAS-PL scale in a group of Polish students of social sciences and humanities. Cronbach’s alpha coefficient had a similar value (α = 0.881), which proves the high internal consistency of the tool, i.e., the high reliability of the questionnaire. The reliability coefficient also exceeded 0.8 in each of the subscales. The verification of the questionnaire also confirmed the compliance of the three-factor structure with the original version of the questionnaire [17].

In Poland, studies on attitudes towards people with disabilities most often involved students of medical and health sciences but less frequently students of other faculties [25,26,27]. The surveyed students of medical faculties declared their readiness to help the disabled. As indicated by the respondents, the disabled should marry and have children, work, and learn in integration classes [25,26]. The first studies based on the MAS-PL scale involved students of medical and health sciences as well [18,19]. Therefore, the authors of the article decided to conduct the survey in a group of students of social sciences and humanities.

In the present study, the respondents achieved a similar mean global score in the MAS-PL scale to that obtained by the Polish students of medicine and health sciences (82.79 and 80.15 points, respectively) [19]. In both studies, there were no global scores close to the maximum possible, which would have indicated extremely negative attitudes towards people with disabilities. The analysis of the mean results in each of the three subscales (affective, cognitive, and behavioural) revealed very similar results in both groups of students [19]. In both studies, the greatest variation in the answers was found in the case of the affective subscale. A difference between the studies was observed in relation to subscales characterised by the lowest differentiation of responses, i.e., the behavioural subscale in the present study, and particularly strong concentration of scores was noted in the cognitive subscale in the case of the students of medical and health sciences [19]. In both studies, there were no differences associated with the analysed majors.

### 4.1. Attitudes towards Disabled People versus Socio-Demographic Variables

Wang et al. conducted a review of publications analysing factors related to the attitudes of society towards people with disabilities. They analysed articles available in three databases (Medline, EMBASE, and Cochrane) published from 1950 to the present time. The review analysed only quantitative studies using valid measurements, and the methodological quality of the included studies was appraised based on three criteria (sample, measurement, and analysis). It was found that sex, age, and education were the most frequently analysed demographic factors influencing attitudes towards people with disabilities [28].

#### 4.1.1. Attitudes towards Disability versus Respondents’ Sex

As shown by the literature review, the sex variable often differentiates declared attitudes. In general, females have a more positive attitude towards people with disabilities than males [28,29,30,31,32,33,34,35,36,37,38,39,40]. Similar relationships were observed in Polish studies, e.g., the MAS-PL study conducted by Radlińska et al. [19]. As reported by Boryszewski, females clearly dominated over males in the declaration of their willingness to help people with disabilities [41]. A study carried out by Gorczycka demonstrated that females exhibited a much better attitude towards the disabled than males. Females more often showed kindness and understanding [23]. Based on their research results, Machalski et al. indicated that, in comparison with males, females were more convinced that disabled people are subject to social discrimination and that it is worth helping and providing them with special care and assistance [42].

Some studies showed no relationship between respondents’ sex and attitudes towards people with disabilities [43,44,45]. Similarly, the global MAS-PL score in the present study did not indicate any significant differences in the attitudes depending on sex. However, such a differentiation was observed in relation to the three analysed components of attitude. The use of the adapted MAS scale, which is a multidimensional tool differentiating the three attitude components (affective, cognitive, and behavioural), provided more detailed knowledge of the declared attitudes than information that would have been provided by one-dimensional analysis. As in the surveys for students of medicine and health sciences [19], the female student exhibited more positive attitudes in terms of the cognitive and behavioural components. The present study also revealed a significant variation with regard to the affective component, in which the males exhibited more positive attitudes. Such a relationship was not reported by Radlińska et al. [19].

In the first MAS-based studies conducted among Jewish Israelis, an analysis of the differences between males and females showed a significant difference in the behavioural subscale, in which the females displayed more positive attitudes than the males [17]. Subsequent studies conducted in a group of Jewish Israelis with the use of revised MAS demonstrated that females displayed more positive attitudes in terms of the cognition subscale [46]. In studies from Serbia, females exhibited more positive attitudes in both the cognitive subscale and approaching behaviour. More negative emotions and avoidance behaviours were exhibited by males. It was also observed that males experienced overtly negative emotions such as disgust, indifference, and the feeling of guilt more often than females [47].

#### 4.1.2. Attitudes towards Disability versus Respondents’ Age

The review of publications analysing factors related to the attitudes of society towards people with disabilities carried out by Z. Wang et al. included papers on the relationship between respondents’ age and declared attitudes. The analysis showed inconsistency in the results. Nine of the 11 analysed studies reported significant differences in attitudes depending on age, but their results were contradictory. In four studies, older people displayed a more positive attitude towards the disabled than younger respondents, while the opposite was found in the other five studies. The observed inconsistency in the results may have been related to the specific characteristics of the respondents in the studies. Additionally, there may have been an effect of other uncontrolled factors, e.g., knowledge of people with disabilities or contact with such persons [28].

Studies conducted in the general population in Serbia aged 18–70 years (N = 2331) with the use of an adaptation of the MAS scale showed no significant correlations between the attitudes towards people with physical disabilities and the age of the respondents [47]. No relationship between age and attitudes towards the disabled was found in the present study and the study on Polish students of medical and health sciences [19]. It should be noted, however, that the respondent group in both cases consisted mainly of young people and did not vary in terms of age. Correlations with age were found in the first MAS scale studies. Although the surveyed group was also not highly diverse in terms of this variable (the research group consisted mainly of Israeli students), more positive attitudes towards people with disabilities in the affective and behavioural subscales were declared by older respondents [17].

#### 4.1.3. Attitudes towards Disability versus Respondents’ Education Level

As shown by the literature review conducted by Wang et al., most of the reviewed publications indicated either a positive correlation between the level of education and attitudes or no such relationship. People with higher education levels were found to be more liberal and open-minded with understanding for people with disabilities and related issues, hence their better attitude towards the disabled [28].

In most of the investigations carried out with the use of the MAS scale, including the first MAS studies conducted in Israel [17], the respondents were mainly students [18,19,46,48]. In studies on a group of Jewish Israelis dominated by students, no differentiation of attitudes depending on education was found [17]. This variable was not analysed in the present study due to the high homogeneity of the level of education in the respondent group. Nevertheless, the intensity of the global and affective scales slightly increased with the year of study. More negative attitudes were also observed in surveys of Greek nursing students from higher years of study. The mean MAS total score and the affective and cognitive subscales were significantly higher in the group of the 7th than 1st semester [48].

#### 4.1.4. Attitudes towards Disability versus the Place of Residence

The present study showed no correlation between students’ attitudes towards the disabled and the place of residents (village or city). Similarly, no such relationships were found in the survey of Polish students of medical and health sciences [19]. Currently, Polish countryside is changing in terms of the social, economic, and cultural aspects, which is reflected in the changing attitudes of the inhabitants of villages and cities. The living conditions in the countryside are similar to those in the city. Not only farmers but also people with higher education levels working in non-agricultural sectors live in the countryside; this phenomenon is referred to as disagrarisation of rural areas. The mosaic of rural lifestyles induces changes in the life profiles of village inhabitants and contributes to openness to social and cultural life, which was previously characteristic for the urban lifestyle [49,50,51]. These changes obliterate the differences in the social attitudes declared by residents of villages and cities, which explains the absence of differences in social attitudes towards the disabled.

### 4.2. Limitations

The present results should be analysed taking into account several limitations. All the respondents were students; therefore, they formed a fairly homogeneous group, which was reflected in the relatively small deviations from the mean results. The surveyed group is not representative of other parts of the population, mainly in terms of age and education. However, it represents a population of students with a large diversity in terms of the majors of social sciences and humanities, which was the authors’ assumption. Since the students represent one university, the results cannot be generalised to all students of social sciences and humanities. It should be noted that the responses are declarative and may in fact be different.

## 5. Conclusions

The analyses confirmed the high reliability of the MAS-PL scale, and the results indicated that students of social sciences and humanities displayed similar attitudes towards the disabled to those declared by Polish students of medicine and health sciences [19]. The absence of differences may result from the process of education of students of humanities, social sciences, and medical and health sciences. The curricula of these majors include modules and courses covering a broad approach to human sciences, taking into account personalistic concepts. Young people who choose these majors exhibit an attitude of openness to other people and willingness to establish social relationships.

The global MAS-PL score showed no significant differences between the sexes. However, such correlations were observed in the case of the three analysed subscales. The female respondents exhibited more positive attitudes towards the disabled in the cognitive and behavioural subscales, while the males displayed more positive attitudes in the affective component. There was no significant effect of the major, age, or place of residence of the respondents. A slight increase in negative attitudes was observed with the year of study (mainly in the affective subscale).

Since the study group is not representative, an interesting idea for further research would be to prepare a survey for a representative group of Polish students with a focus on majors and socio-demographic features. It is also worth including a representative group of students of sciences and engineering-technical faculties.

Non-acceptance for the participation of the disabled in social interactions increases the social distance and the feeling of embarrassment or even fear of direct contact with such persons. Therefore, the authors recommend initiating activities, projects, and classes aimed at introducing students to the issues of functioning of people with disabilities. Conscious and intentional dissemination of knowledge of people with disabilities should not focus on their limitations but on their activity and participation in social life. An increase in the variety of forms of social integration seems to be of key importance. Staying in the same physical space does not guarantee elimination of social attitudes of rejection or avoidance. There must be a space for psychological and social integration, which means the coexistence of students with disabilities in all social systems at universities. The university can ensure activation of disabled people in student councils by promotion of lifestyles providing room for students with disabilities. As part of these activities, students with disabilities should be engaged in participation in social life by attendance at concerts and going to the swimming pool, gym, and trips. In this way, students will have the opportunity to integrate and overcome barriers of exclusion and social stigma. Students are the group that will constitute social elites deciding about the future. The 21st century society should be ready to adopt attitudes of tolerance of differences and inclusion of people with dysfunctions.

## Figures and Tables

**Table 1:** Characteristics of the respondents (*n* = 540).

Variable	*n*	%
Sex		
Female	366	67.8
Male	174	32.2
Place of residence		
city	263	48.7
village	277	51.3
Year of study		
1st	247	45.7
2nd	149	27.6
3rd or higher	144	26.7
Major		
National Security	113	20.9
Economics	83	15.4
Pedagogy	95	17.6
Finance and Accounting	88	16.3
Management	52	9.6
Philology	69	12.8
Sociology	40	7.4

**Table 2:** Principal component factor analyses of the MAS-PL scale (Varimax rotation and Cronbach’s alpha).

MAS Factors	Affects	Cognition	Behaviours
He/she looks friendly (cognition 4)	0.853		
We may get along really well (cognition 3)	0.847		
I enjoy meeting new people (cognition 5)	0.808		
I can always talk with him/her about things that interest both of us (cognition 7)	0.808		
He/she looks like an OK person (cognition 2)	0.805		
Why not get to know him/her better? (cognition 9)	0.788		
I can make him/her feel more comfortable. (cognition 8)	0.766		
He/she will enjoy getting to know me (cognition 6)	0.749		
He/she seems to be an interesting boy/girl (cognition 1)	0.746		
Start a conversation (behaviour 8)	0.653		0.167
He/she will appreciate it if I start a conversation (cognition 10)	0.611	−0.140	
Initiate a conversation if he/she doesn’t make the first move (behaviour 7)	0.539		0.126
Serenity (emotion 7)	0.520	0.304	−0.160
Calmness (emotion 8)	0.374	0.478	−0.269
Alertness (emotion 16)	−0.318	0.164	0.263
Pity (emotion 14)	−0.302	0.374	0.128
Stress (emotion 2)		0.802	0.102
Nervousness (emotion 4)		0.791	0.157
Upset (emotion 11)		0.789	0.251
Fear (emotion 10)		0.732	0.197
Tension (emotion 1)		0.713	
Helplessness (emotion 3)		0.683	0.207
Shame (emotion 5)		0.667	0.137
Shyness (emotion 13)	−0.147	0.588	0.140
Depression (emotion 9)		0.544	0.308
Guilt (emotion 12)		0.486	0.327
Disgust (emotion 15)	0.226	0.381	0.456
Relaxation (emotion 6)	0.278	0.332	−0.319
Get up and leave (behaviour 2)	0.166	0.162	0.756
Move to another table (behaviour 6)	0.167	0.164	0.732
Read the newspaper or talk on a cell phone (behaviour 3)		0.190	0.728
Find an excuse to leave (behaviour 5)		0.284	0.705
Move away (behaviour 1)		0.297	0.661
Continue what he/she was doing (behaviour 4)	−0.264	0.127	0.478
Variance explained	7.717	5.784	3.781
Cronbach’s alpha	0.898	0.871	0.840

Loadings of negligible strength (<0.1) were hidden.

**Table 3:** Basic distribution of MAS-PL scores in the survey (N = 540).

MAS-PL	Possible Range	Min–Max	M	SD	Skewness	Kurtosis	S-W Test
Global score	34–170	39–136	82.79	16.33	−0.022	−0.127	0.3752
Affective subscale	16–80	18–68	40.18	10.04	0.227	−0.566	0.0002
Cognitive subscale	10–50	10–50	24.29	8.18	0.254	−0.387	<0.0001
Behavioural subscale	8–40	8–36	18.31	5.30	0.388	−0.099	<0.0001

Min—minimum; Max—maximum; M—mean; SD—standard deviation; S-W test—Shapiro-Wilk test of normality.

**Table 4:** Relationships between the results of the individual subscales and the global MAS-PL scale.

MAS-PL	Global Score		Affective Subscale		Cognitive Subscale		Behavioural Subscale	
	r_s_	*p*	r_s_	*p*	r_s_	*p*	r_s_	*p*
Global score	-	-	0.75	<0.0001 *	0.58	<0.0001 *	0.71	<0.0001 *
Affective subscale	0.75	<0.0001 *	-	-	0.04	0.9620	0.43	<0.0001 *
Cognitive subscale	0.58	<0.0001 *	0.04	0.9620	-	-	0.25	<0.0001 *
Behavioural subscale	0.71	<0.0001 *	0.43	<0.0001 *	0.25	<0.0001 *	-	-

r_s_—Spearman’s rank-order correlation; *—significant correlation at *p* < 0.05.

**Table 5:** MAS-PL scale results relative to the majors.

Major		Global Score		Affective Subscale		Cognitive Subscale		Behavioural Subscale	
	*n*	Mean	SD	Mean	SD	Mean	SD	Mean	SD
National Security	113	79.53	13.37	38.46	9.14	23.61	7.84	17.46	4.75
Finance and Accounting	88	82.16	15.88	40.78	10.28	23.17	7.16	18.20	5.68
Economics	83	82.82	17.29	41.25	10.53	22.84	7.30	18.72	5.11
Management	52	83.29	20.68	37.79	9.82	26.27	9.65	19.23	6.20
Pedagogy	95	83.94	15.79	41.60	9.04	24.15	7.95	18.19	5.22
Philology	69	84.88	15.91	40.25	11.04	26.01	9.40	18.62	5.60
Sociology	40	86.33	17.92	41.13	11.11	26.50	8.30	18.70	4.64
Total	540	82.79	16.33	40.18	10.04	24.29	8.18	18.31	5.30

SD—standard deviation.

**Table 6:** MAS-PL scores relative to respondents’ sex.

MAS-PL	Man		Woman		Total		Mann-Whitney U Test	
	Mean	SD	Mean	SD	Mean	SD	Z	*p*
Global score	83.63	15.68	82.39	16.63	82.79	16.3	0.85	0.3948
Affective subscale	38.36	9.92	41.05	9.99	40.18	10.0	−2.77	0.0056 *
Cognitive subscale	26.06	7.49	23.45	8.37	24.29	8.2	3.64	0.0003 *
Behavioural subscale	19.21	5.19	17.89	5.30	18.31	5.3	3.09	0.0020 *

SD—standard deviation; *—significant differences at *p* < 0.05.

**Table 7:** Relationships between the results of the MAS-PL scales and the age and year of study of the surveyed students.

MAS-PL	Age		Year of Study	
	r_s_	*p*	r_s_	*p*
Global score	−0.01	0.8387	0.12	0.0061 *
Affective subscale	0.04	0.3110	0.19	<0.0001 *
Cognitive subscale	−0.07	0.0978	−0.03	0.4670
Behavioural subscale	−0.03	0.4513	0.07	0.0828

r_s_—Spearman’s rank-order correlation; *—significant correlation at *p* < 0.05.

**Table 8:** Results of the MAS-PL subscales relative to the place of residence.

MAS-PL	City		Village		Total		Mann-Whitney U Test	
	Mean	SD	Mean	SD	Mean	SD	Z	*p*
Global score	82.94	16.74	82.64	15.95	82.79	16.33	−0.09	0.9270
Affective subscale	40.57	9.91	39.81	10.17	40.18	10.04	0.91	0.3631
Cognitive subscale	24.03	8.40	24.54	7.97	24.29	8.18	−0.99	0.3237
Behavioural subscale	18.34	5.40	18.29	5.21	18.31	5.30	0.12	0.9017

SD—standard deviation.

## Data Availability

The data presented in this study are on request available from the corresponding author.

## References

[1] World Health Organization https://www.who.int/health-topics/disability#tab=tab_1.

[2] Główny Urząd Statystyczny (GUS) (2012). Raport z Wyników. Narodowy Spis Powszechny Ludności i Mieszkań 2011.

[3] Trębicka-Postrzygacz B. (2017). About disability in definitions and regulations in the perspective of social inclusion. Disabl. Stud. Sketches Thesis.

[4] World Health Organization (2001). International Classification of Functioning, Disability and Health.

[5] United Nations General Assembly (2006). Convention on the Rights of Persons with Disabilities, Resolution Adopted by the General Assembly United Nations on 13 December 2006.

[6] Ustawa z dnia 15 Czerwca 2012 r. o Ratyfikacji Konwencji o Prawach osób Niepełnosprawnych, Sporządzonej w Nowym Jorku dnia 13 Grudnia 2006 r. Dziennik Ustaw Rzeczypospolitej Polskiej, Warszawa, dnia 2 Sierpnia 2012, poz. 882. http://isap.sejm.gov.pl/isap.nsf/download.xsp/WDU20120000882/O/D20120882.pdf.

[7] Konwencja o Prawach osób Niepełnosprawnych, Sporządzona w Nowym Jorku dnia 13 Grudnia 2006 r. Dziennik Ustaw Rzeczypospolitej Polskiej, Warszawa, dnia 25 Października 2012 r., poz. 1169. http://isap.sejm.gov.pl/isap.nsf/download.xsp/WDU20120001169/O/D20121169.pdf.

[8] Rao S. (2004). Faculty attitudes and students with disabilities in higher education: A literature review. Coll. Stud. J..

[9] Suria-Martinez R. (2011). Análisis comparative osobre las actitudes de los estudiantes hacia sus compañeros con discapacidad. Electron. J. Res. Educ. Psychol..

[10] Tomczyszyn D., Leszczyńska-Rejchert A., Kantowicz E. (2012). Stereotypy społeczne jako społeczne nastawienia wobec osób z niepełnosprawnością intelektualną. Stereotypy a Starość i Niepełnosprawność.

[11] Girli A., Sarı H.Y., Kırkım G., Narin S. (2016). University students’ attitudes towards disability and their views on discrimination. Int. J. Dev. Disabil..

[12] Tomczyszyn D. (2020). Social and vocational rehabilitation of people with disabilities. Soc. Diss..

[13] Aronson E., Wilson T.D., Akert R.M. (1997). Psychologia Społeczna. Serce i Umysł.

[14] Major M., Ulman P. (2011). Charakterystyka wybranych postaw społecznych w Polsce. Analiza statystyczna. Crac. Rev. Econ. Manag..

[15] Tuczyński K., Walat W. (2019). Trójczynnikowa koncepcja postawy człowieka wobec wykorzystania e-learningu w procesie kształcenia. Educ. -Technol. -Comput. Sci..

[16] Rogozińska-Pawełczyk A. (2014). Kształtowanie postawy zaangażowania organizacyjnego. Hum. Resour. Manag..

[17] Findler L., Vilchinsky N., Werner S. (2007). The multidimensional Attitudes Scale toward persons with disabilities (MAS): Construction and Validation. Rehabil. Couns. Bull..

[18] Radlińska I., Starkowska A., Kożybska M., Flaga-Gieruszyńska K., Karakiewicz B. (2020). The multidimensional attitudes scale towards persons with disabilities (MAS)—A Polish adaptation (MAS-PL). Ann. Agric. Environ. Med..

[19] Radlińska I., Kożybska M., Karakiewicz B. (2021). Attitudes of Polish Medical and Health Sciences Students towards Persons with Physical Disabilities Using the MAS-PL Scale. Int. J. Environ. Res. Public Health.

[20] Satchidanand N., Gunukula S.K., Lam W.Y., McGuigan D., New I., Symons A.B., Withiam-Leitch M., Akl E.A. (2012). Attitudes of healthcare students and professionals toward patients with physical disability: A systematic review. Am. J. Phys. Med. Rehabil..

[21] Ostrowska A. (2015). Stosunek społeczeństwa do osób z niepełnosprawnością na podstawie badań z lat 1993–2013: Jak postępuje proces integracji?. Sci. Issues Health Prot..

[22] Nowak K. (2015). Postawy młodzieży wobec osób niepełnosprawnych ruchowo. Ann. Uniwersitatis Maria Curie Skłodowska Lub. –Pol..

[23] Gorczycka E., Lecewicz-Bartoszewskiej J., Polak-Sopińska A. (2011). Z badań nad postawami wobec osób niepełnosprawnych. Ergonomia Niepełnosprawnym—Współczesne i Przyszłe Kierunki Rozwoju.

[24] Kazanowski Z. (2019). Postawy wobec integracji społecznej osób z niepełnosprawnością w kontekście zmiany pokoleniowej. Educ.-Technol.-Comput. Sci..

[25] Krajewska-Kułak E., Kułak W., Łukaszuk C., Lewko J., Lankau A., Damme-Ostapowicz K., Rozwadowska E., Kropiwnicka E., Guzowski A. (2011). Postawy studentów kierunku pielęgniarstwo wobec osób niepełnosprawnych. Hygeia Public Health.

[26] Żebrowska J.M., Krajewska-Kułak E., Łukaszuk C. (2012). Postawy studentów wydziału nauk o zdrowiu wobec osób niepełnosprawnych. Nurs. Probl..

[27] Wiliński W., Świercze R., Wieczorek M. (2013). Postawy studentów AWF we Wrocławiu wobec osób z niepełnosprawnością intelektualną. Sci. Treatises Univ. Sch. Phys. Educ. Wroc..

[28] Wang Z., Xu X., Han Q., Chen Y., Jiang J., Ni G.X. (2021). Factors associated with public attitudes towards persons with disabilities: A systematic review. BMC Public Health.

[29] Paris M.J. (1993). Attitudes of medical students and health-care professionals toward people with disabilities. Arch. Phys. Med. Rehabil..

[30] Forlin C., Fogarty G., Carroll A. (1999). Validation of the factor structure of the Interactions with Disabled Persons Scale. Aust. J. Psychol..

[31] Werner P., Davidson M. (2004). Emotional reactions of lay persons to someone with Alzheimer’s disease. Int. J. Geriatr. Psychiatry.

[32] Laws G., Kelly E. (2005). The attitudes and friendship intentions of children in United Kingdom mainstream schools toward peers with physical or intellectual disabilities. Int. J. Disabil. Dev. Educ..

[33] Panek P.E., Smith J.L. (2005). Assessment of terms to describe mental retardation. Res. Dev. Disabil..

[34] Siperstein G.N., Parker R.C., Bardon J.N., Widaman K.F. (2007). A national study of youth attitudes toward the inclusion of students with intellectual disabilities. Except. Child..

[35] Panek P.E., Jungers M.K. (2008). Effects of age, gender, and causality on perceptions of persons with mental retardation. Res. Dev. Disabil..

[36] Sahin H., Akyol A.D. (2010). Evaluation of nursing and medical students’ attitudes towards people with disabilities students’ attitudes towards people with disabilities. J. Clin. Nurs..

[37] Bossaert G., Colpin H., Pijl S.J., Petry K. (2011). The attitudes of Belgian adolescents towards peers with disabilites. Res. Dev. Disabil..

[38] Goreczny A.J., Bender E.E., Caruso G., Feinstein C.S. (2011). Attitudes toward individuals with disabilities: Results of a recent survey and implications of those results. Res. Dev. Disabil..

[39] de Laat S., Freriksen E., Vervloed M.P.J. (2013). Attitudes of children and adolescents toward persons who are deaf, blind, paralyzed Or intellectually disabled. Res. Dev. Disabil..

[40] Patka M., Keys C.B., Henry D.B., McDonald K.E. (2013). Attitudes of Pakistani community members and staff toward people with intellectual disability. Am. J. Intellect. Dev. Disabil..

[41] Boryszewski P. (2007). Niepełnosprawni w Opinii Społeczności Lokalnych na Przykładzie 10 Wybranych Gmin w Polsce.

[42] Machalski D., Kołpa M., Grochowska A. (2019). Postrzeganie osób niepełnosprawnych w społeczeństwie. Health Promot. Phys. Act..

[43] Shiloh S., Heruti I., Berkovitz T. (2011). Attitudes toward people with disabilities caused by illness or injury: Beyond physical impairment. Int. J. Rehabil. Res..

[44] Vincent-Onabajo G.O., Malgwi W.S. (2015). Attitude of physiotherapy students in Nigeria toward persons with disability. Disabil. Health J..

[45] Ruiz P.O., Gonzalez-Medina G., Couso A.S., Palomares M.J., Mansilla J.R., Ardila E.M.G., Vicente M.N.M. (2020). Attitude towards People with Disability of Nursing and Physiotherapy Students. Children.

[46] Vilchinsky N., Werner S., Findler L. (2010). Gender and attitudes toward people using wheelchairs: A multidimensional perspective. Rehabil. Couns. Bull..

[47] Dragojević N., Milačić-Vidojević I., Hanak N. (2010). Attitudes toward People with Physical Disabilities, their structure and correlating variables. Spec. Educ. Rehabil. Sci. Pract..

[48] Polikandrioti M., Govina O., Vasilopoulos G., Adamakidou T., Plakas S., Kalemikerakis I., Galanis P., Fouka G. (2020). Nursing Students’ Attitudes towards People with Disabilities. Int. J. Caring Sci..

[49] Podedworna H., Łaciak B. (2010). Styl życia mieszkańców wsi. Polskie Style Życia. Między Miastem a Wsią. Kongres Obywatelski.

[50] Rewers E., Łaciak B. (2010). W poszukiwaniu polskiej miejskości, czyli na czym polega specyfika naszej klasy Kreatywnej?. Polskie Style Życia. Między Miastem a Wsią. Kongres Obywatelski.

[51] Halamska M. (2013). Życie na wsi: Elementy stylu życia. Village Agric..

